# Should we treat pyrexia? And how do we do it?

**DOI:** 10.1186/s13054-016-1467-2

**Published:** 2016-10-03

**Authors:** James F. Doyle, Frédérique Schortgen

**Affiliations:** 1 Department of Intensive Care Medicine and Surrey Peri-Operative Anaesthesia and Critical Care Collaborative Research Group, Intensive Care Unit, Royal Surrey County Hospital NHS Foundation Trust, Egerton Road, Guildford, GU2 7XX Surrey UK; 2 Service de Réanimation Médicale, Groupe Hospitalier Henri Mondor-APHP, 94000 Créteil, France

## Abstract

The concept of pyrexia as a protective physiological response to aid in host defence has been challenged with the awareness of the severe metabolic stress induced by pyrexia. The host response to pyrexia varies, however, according to the disease profile and severity and, as such, the management of pyrexia should differ; for example, temperature control is safe and effective in septic shock but remains controversial in sepsis. From the reported findings discussed in this review, treating pyrexia appears to be beneficial in septic shock, out of hospital cardiac arrest and acute brain injury.

Multiple therapeutic options are available for managing pyrexia, with precise targeted temperature management now possible. Notably, the use of pharmacotherapy versus surface cooling has not been shown to be advantageous. The importance of avoiding hypothermia in any treatment strategy is not to be understated.

Whilst a great deal of progress has been made regarding optimal temperature management in recent years, further studies will be needed to determine which patients would benefit the most from control of pyrexia and by which means this should be implemented. This narrative review is part of a series on the pathophysiology and management of pyrexia.

## Background

Around 35 % of in-hospital patients will develop pyrexia [1], increasing up to 70 % amongst the critically unwell [2]. Pyrexia has long been thought of as a protective physiological response to help host defences, although this is now being challenged. Despite recent advances, it remains unclear whether pyrexia or the physiological response to pyrexia causes morbidity and mortality and whether management of pyrexia with pharmacological agents or physical cooling actually confers benefit. We review some of the recent evidence for and against treating pyrexia with reference to varying disease severity. Finally, we discuss treatment strategies and methods.

This narrative review of pyrexia and associated treatment options is based on the latest available published evidence. We searched MEDLINE, EMBASE and CINAHL for articles published in English before 12 Feb 2016. We used the search terms “fever”, “pyrexia”, “hyperthermia” in combination with “ICU” or “sepsis” or “brain injury” or “cardiac arrest” and with “cooling” or “antipyretics” or “acetaminophen” or “NSAIDS”. We largely selected publication from the past 15 years. Further evidence was selected from these articles’ reference lists and from our previous knowledge of the subject. Review articles are cited to provide further information on aspects that are not within the remit of this article.

## What is pyrexia?

### Pathophysiology

The process of tightly regulating body temperature within a specified range (±0.2 °C), or thermoregulation, is an essential homeostatic mechanism. Thermoregulation consists of afferent signalling via warm and cold thermoreceptors, central processing within the hypothalamus and efferent response. These responses include regulation of peripheral blood flow, diaphoresis and shivering. Whilst there is strict control there is also rhythmic temperature variability over a 24-h period [3]. This circadian rhythm is altered in critically ill patients with both temporal shifts and a larger magnitude of variation, both increasing with disease severity [4].

**Fig. 1:**
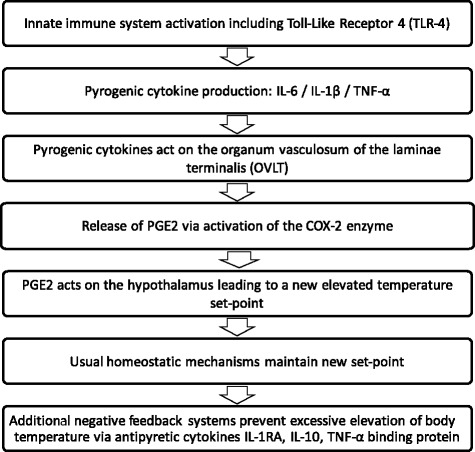
The main stages of the molecular basis of pyrexia. *IL* interleukin, *PGE2* prostaglandin E2, *TNF* tumour necrosis factor

Pyrexia (also named fever) is the altering upward of the thermoregulatory set point, often secondary to the systemic inflammatory response to a stimulus such as infection. The molecular basis is summarized in Fig. 1 [5, 6]. Fever has been defined by The American College of Critical Care Medicine, the International Statistical Classification of Diseases and the Infectious Diseases Society of America as a core temperature of 38.3 °C or higher [7]. Pyrexia secondary to the systemic inflammatory response should be distinguished from hyperthermia resulting from excessive heat production, as observed in heatstroke and malignant syndromes, or from ineffective heat loss. Temperature levels encountered during hyperthermia are usually higher than during pyrexia because thermoregulation is abolished; indication of rapid temperature control is, therefore, indisputable to avoid irreversible tissue damage.

### Grading and measurement

The definition of pyrexia in itself is complex as there is no agreed consensus. This is further complicated by peripheral thermometers not accurately estimating body core temperature [8]. The causes of pyrexia are multiple and contribute to different definitions. During infection, fever is usually defined as a temperature greater that 38.3 °C [7, 9]; in the post-resuscitation care of cardiac arrest, a threshold of 37.6 °C is used [10]; and in stroke, thresholds of 37.2, 37.5 and 38 °C are all applied [11]. Whatever the clinical situation, hypothermia is usually defined by a core temperature lower than 36 °C [7, 10, 12].

## Should we treat pyrexia?

### The cost of pyrexia

The cost of pyrexia should be considered in several ways. Pyrexia has a metabolic cost such that cooling febrile ICU patients will reduce oxygen consumption by 10 % per °C [6]. Small studies in sedated patients demonstrated a significant reduction in VO_2_ (the rate of oxygen consumption) and VCO_2_ (the rate of carbon dioxide elimination ) during cooling [13, 14]. In septic shock, temperature lowering by ibuprofen was associated with increased lactate clearance [15]. In patients with acute brain injury, pyrexia may increase intracranial pressure and worsen secondary ischemic damage [16]. These suggest the possibility of therapeutically offloading the cardiorespiratory system and preserving brain function at times of stress. Whether the cost of pyrexia translates to unfavourable outcomes remains unknown. The incidence of pyrexia is decreasing over time with an absolute reduction of 35 % found in Canadian ICUs [17]. This did not coincide with an appreciable decrease in mortality, suggesting that important outcomes may not be affected by the incidence of pyrexia.

**Fig. 2:**
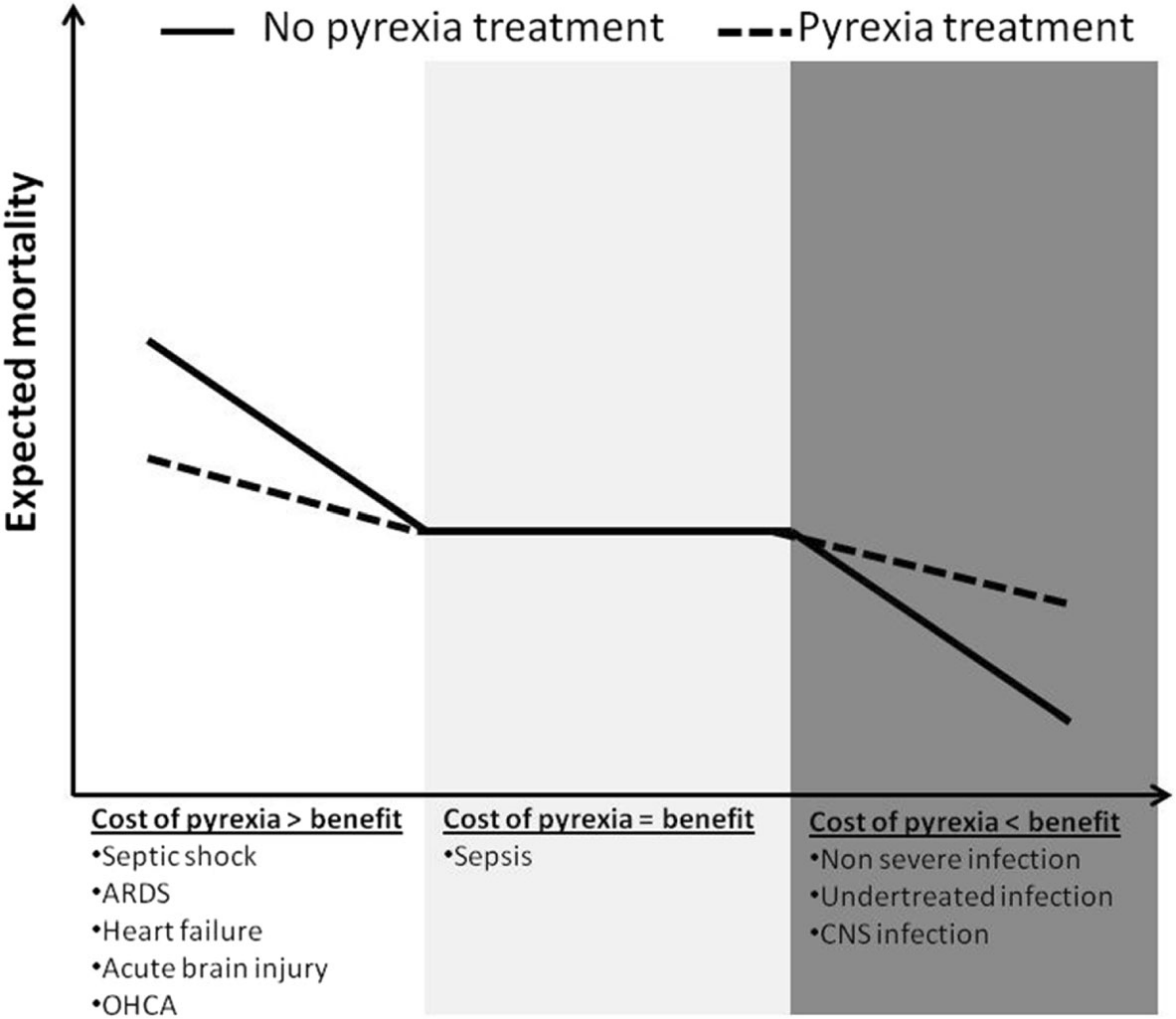
Suggested impact of pyrexia treatment on outcome according to clinical context. *ARDS* acute respiratory distress syndrome, *CNS* central nervous system, *OHCA* Out-of-hospital cardiac arrest

Perhaps the question should not be “should we treat pyrexia?” but “in what conditions is it beneficial to treat pyrexia?” (Fig 2). This is highlighted in a large observational study where fever within the first 24 h of ICU admission was significantly associated with decreased mortality in patients with infection while peak fever ≥40 °C was associated with increased mortality in patients without infection [18]. An observational study on 1400 non-neurological critically ill patients also revealed different associations between the maximal peak temperature and mortality according to the presence of sepsis or not [19]. Fever ≥39.5 °C was associated with increased mortality in non-septic patients while moderate fever (37.5–38.4 °C) was associated with decrease mortality in septic patients. Moreover, this study highlights different impacts of fever treatment. Physical cooling did not alter the mortality risk and the use of antipyretic agents did not alter mortality in the non-septic group but did increase 28-day mortality in the septic group (adjusted odds ratio 2.61 (*P* = 0.028) for non-steroidal anti-inflammatory drugs (NSAIDs) and 2.05 (*P* = 0.01) for paracetamol [19].

In patients with acute brain injury, pyrexia has been identified as an independent risk factor for increased mortality and poorer neurological outcome [16, 20–22]. Results are, however, inconsistent as fever could be a marker of brain injury severity [23]. The presence or not of infection may also alter the relationship between body temperature and outcome [24, 25]. In more than 100,000 patients, a negative association between early peak fever above 39 °C and hospital mortality was found in patients with traumatic brain injury and stroke but not in patients with central nervous system infection [25].

Pyrexia related to whole body ischemia-reperfusion syndrome is frequent after cardiac arrest and studies found a negative impact of pyrexia on mortality [10]. Patients with successful return of spontaneous circulation are considered as good candidates for targeted temperature management (TTM) with the minimal goal of not exposing patients to pyrexia [10].

Besides the context of fever and disease severity, individual patient’s characteristics may determine the ability to cope with the cost of pyrexia, costs that may be detrimental in those patients with low cardiac and/or respiratory reserve, typically seen in elderly patients and those with significant comorbidities. Evidence to quantify this in terms of the ability to cope with the cost of pyrexia is not available, so consideration of the clinical context is required.

### Pyrexia treatment in specific situations

#### Sepsis

For many years pyrexia has been considered a physiological host defence which may confer benefit. The development of antipyretics was justified in terms of patient comfort and the physiological reduction of cardiorespiratory stress. During sepsis, fever is not consistently reported as deleterious and may be protective [18, 19]. The opposite impacts of pyrexia on inflammation and microbiological control may explain discrepant results. Pyrexia enhances inflammation but decrease bacterial and viral load. This dual effect has been highlighted in animals with pneumonia, showing that pyrexia treatment is beneficial for survival only when antibiotics allow effective control of bacterial load [26].

Whilst the advantages of cooling in sepsis remain a controversial topic, there is now good evidence that cooling is safe and effective in septic shock. One study allocated 200 sedated and ventilated patients with severe sepsis on vasopressors to external cooling or none [27]. The findings demonstrated a significant decrease in vasopressor requirement and in 14-day mortality (19 versus 34 %; absolute difference −16 %; 95 % confidence interval (CI) −28 to −4) with cooling. The mortality outcome was similar. In a post hoc analysis, it was confirmed that temperature control was a main mediator of early mortality [28]. The benefits of cooling could be explained by specific patients’ profiles and the cooling strategy used. Patients with severe sepsis benefit the most from the prevention of pyrexia. In this trial the main source of infection was pneumonia with a large majority of patients under adequate antimicrobial therapy at the time of cooling initiation. None were exposed to hypothermia and only two experienced shivering, all being sedated.

The “HEAT” study compared pyrexia control by intravenous paracetamol with placebo in 691 randomized ICU patients with suspected infection and temperature >38 °C [29]. Only 20 % of patients experienced septic shock. Paracetamol was well tolerated. The outcomes for ICU free days and 28-day and 90-day mortalities were similar between the groups.

These two randomised controlled trials (RCTs) in sepsis show that fever control is safe. Interestingly, both noted that patients who received pyrexia treatment had a longer time to death. The avoidance of pyrexia costs at the early stage of severe infection may be balanced by delayed adverse effects. Of these, an acquired hypo-immune state may lead to increased late mortality.

#### Out of hospital cardiac arrest

Out of hospital cardiac arrest (OHCA) is one of the most studied areas for the practical application of temperature control in adults. The physiological basis of cooling management strategies is relevant [10]. Hypothermia reduces cerebral metabolism, inflammation and cell death. These favourable effects resulted in improved neurological outcome of comatose patients with shockable rhythm managed with 32–34 °C TTM [30]. Of note, pyrexia was not treated in the control group; thus, the TTM benefit may have been merely related to the avoidance of pyrexia rather than to hypothermia itself. The latest evidence from this field comes from a large RCT comparing TTM at 33 °C and 36 °C. The benefit seen previously from hypothermia disappeared, with no significant difference in mortality or neurological outcome [31]. This has led to European guidelines changing to indicate a target between 32 and 36 °C for OHCA patients in whom temperature control is used [10]. Whether simple prevention of pyrexia or strict modest hypothermia (36 °C) is required remains to be tested. In children, TTM at 33 °C was compared with normothermia (target 36.8 °C) [32]. Survival and neurobehavioral outcomes were similar, suggesting that a strict avoidance of pyrexia may help minimise secondary brain injury.

#### Acute brain injury

For decades, experts advocated for aggressive treatment of pyrexia in neurological critical care and the pathophysiological basis of secondary brain injury caused by hyperthermia is supported by strong evidence. Pyrexia control is, however, not supported by evidence from clinical comparative studies.

##### Traumatic brain injury

Hyperthermia is common in traumatic brain injury (TBI) and has been shown to worsen neurological outcome. In a comparative cohort study the implementation of strict normothermia via means of intravascular cooling demonstrated a significant reduction of intracranial pressure [33]. Clinical studies have also examined therapeutic hypothermia but failed to demonstrate better outcome, with more frequent favourable neurological outcomes in the normothermia group [34]

##### Cerebrovascular diseases

Treatment of pyrexia is advocated by guidelines for acute stroke management [11]. The largest RCT did not find better neurological outcome with paracetamol therapy initiated within the first 12 h in patients with admission temperatures of 36–39 °C [35]. A post hoc analysis showed a beneficial effect in the group of patients with higher baseline temperature (37–39 °C). A new trial focusing on these patients is on-going [36].

##### Seizure control

Pyrexia decreases the seizure threshold and temperature control is thus advocated in the control of status epilepticus. Although viewed as a good clinical practice, it is not supported by clinical studies.

#### Organ donation

Brain death results in the loss of temperature control. Hyperpyrexia can be encountered initially but hypothermia usually occurs thereafter. Guidelines for organ donor management recommend that physiological parameters, including body core temperature, should be maintained in normal ranges without scientific evidence [37]. Hypothermia could, however, prevent or reduce ischemia-reperfusion injury in several organs. Hypothermia of 34–35 °C compared with normothermia (36.5–37.5 °C) in organ donors has been recently found to significantly reduce the incidence of delayed graft function in kidney recipients [38]. If hypothermia can improve kidney graft functions, it would be justified to, at the least, treat fever. This trial raises the question of the impact of temperature control on acute kidney injury prevention in general, which remains unclear [39].

#### General ICU patients

Many other clinical situations with systemic inflammation or endocrine disorders can promote fever. Since the cause of fever may determine a patient’s outcome by itself, no conclusion can be drawn from observational studies on the impact of fever in general ICU patients. A systematic approach to controlling pyrexia in general ICU patients is not supported by evidence.

### Overall guidance

Several attempts have been made in the literature to discern best practice for pyrexia management in critically ill patients (Table 1). Given the above conflicting data, the association between pyrexia, aetiology, antipyretic management, morbidity, and mortality is particularly complex, with more unanswered questions than answered. As illustrated in Fig. 2, some critically ill patients may benefit from fever control while others may benefit from pyrexia. It is important, however, to put into context the severity of disease; for example, whilst pyrexia may be of benefit in non-severe infection, in a condition with low morbidity and mortality the issue of patient comfort may override any benefit from permissive pyrexia.

**Table 1:** Main RCTs comparing antipyretics with no treatment in adult critically ill patients

	Study	Patients	Number	Temperature criteria at inclusion	Baseline temperature in the treatment group	Antipyretic method	Duration of treatment	Primary end point	Main results
SIRS and Sepsis	Gozolli et al. [41]	SIRS	38	≥38.5 °C	39 (SD 0.3)	Surface cooling	Up to fever resolution (≤37.5 °C)	Temperature difference	Similar temperature and comfort evolution
	Bernard et al. [15]	Severe sepsis	455	None^a^	37.9 (SE 0.2)	NSAID: IV ibuprofen 10 mg/kg/6 h	48 h	30-day mortality	Lower temperature in the treatment group
									No difference in mortality
	Memis et al. [50]	Severe sepsis	40	None^a^	37.8 (SD 0.75)	NSAID: IV lornoxicam 8 mg/12 h	72 h	Anti-inflammatory effects	Similar temperature evolution
	Schortgen et al. [27]	Septic shock	200	≥38.3 °C	38.8 (IQR 38.6–39.2)	TTM 36.5–37 °C with surface cooling	48 h	Dose of vasopressor	Less vasopressor requirement and 14-day mortality in the treatment group
	Janz [48]	Severe sepsis	40	None^a^	37.7 (IQR 37–38.5)	IV paracetamol 1 g/6 h	3 days	Antioxidant effect	Lower maximal temperature in the treatment group
	Young et al. [29]	Suspected infection	700	≥38 °C	38.5 (SD 0.5)	IV paracetamol 1 g/ 6 h	Up to fever resolution (<37.5 °C, 24 h) or day 28	ICU-free days up to day 28.	Lower temperature in the treatment group
									No difference in ICU-free days
Acute brain injury	den Hertog et al. [35]	Stroke	1400	Between 36 and 39 °C	36.9 (SD 0.6)	Enteral paracetamol 1 g/4 h	72 h	Modified Rankin scale at 3 months	Lower temperature in the treatment group
									No difference in neurological outcome
	Saxena et al. [46]	TBI	41	Between 36 and 39 °C	37.3 (SD 0.8)	IV paracetamol 1 g/4 h	72 h	Temperature difference	No difference in temperature

^a^Antipyretics were given with the aim of testing the anti-inflammatory effects of NSAIDs

*IQR* 25th–75th interquartile range, *IV* intravenous, *NSAID* non-steroidal anti-inflammatory drug, *SD* standard deviation, *SE* standard error, *SIRS* systemic inflammatory response syndrome, *TBI* traumatic brain injury

A meta-analysis limited to RCTs of antipyretic therapy in the ICU included five trials totalling 399 patients and did not find a difference in mortality [40]. The inclusion of the more recent “HEAT” study would not change this result [29].

## Management of pyrexia

### Temperature target

Different approaches to fever treatment have been proposed:

- Control of pyrexia when it occurs: treatment administered when temperature exceeds a predefined threshold
- Strict avoidance of pyrexia: temperature maintained below fever threshold
- Strict maintenance of normothermia: TTM with a predefined normothermia range, e.g., 36–37 °C.

The absence of consensus over a definition of fever, the multitude of clinical situations and the scarcity of trials hinder setting goals for clinical practice in terms of treatment timing, rapidity of normothermia induction, temperature target and duration of treatment.

For patients with OHCA, some data can be drawn from the TTM 33 versus 36 °C study [31]. After the 4-h period to achieve the targeted temperature, 95 % of the patients in the 36 °C group had a core body temperature below 37.5 °C for the first 24 h. Treatment of pyrexia in this population may, therefore, correspond to a strict maintenance of body temperature below 37.5 °C. Whether strict normothermia is superior to a strategy that aims to control pyrexia at >37.5 °C once it occurs remains to be tested.

In the “Eurotherm” study, the evolution of body core temperature shows that, in the control group, patients were strictly maintained at 37 °C, which could correspond to “standard” normothermia in TBI [34].

In septic shock, fever control with a TTM of 36.5–37 °C over a 48-h period was found to be advantageous [27]. In a post hoc analysis, the association between different thresholds of temperature and mortality were tested [28]. The time spent with a core body temperature below 38.4 °C within the first 48 h was the most discriminatory. This raises the question of whether a strict avoidance of pyrexia could be sufficient to induce similar benefits.

### Efficacy and risks of antipyretic methods

Antipyretic agents, mainly paracetamol and NSAIDs, and physical cooling methods can be used to control pyrexia. Cooling with surface devices is usually preferred for fever control while endovascular methods are more commonly restricted to therapeutic hypothermia. Infusions of cold fluids are easy to administer and inexpensive but this strategy exposes patients to unnecessary volume expansion and does not allow precise temperature control.

Antipyretic agents act on the hypothalamic set point. To be effective, the integrity of the thermoregulatory system should be intact. This explains why antipyretic agents are usually ineffective in the control of pyrexia in acute brain injury [16]. Cooling reduces temperature by removing heat without decreasing the set point, which exposes patients to reflex shivering. These different mechanisms have opposite consequences on vasotonicity. The fall in temperature set point promotes vasodilation to enhance heat loss whilst cooling induces vasoconstriction. In patients with sepsis, this results in different mean arterial pressure evolution [41].

Methods of temperature management have mostly been studied in the context of hypothermia induction and have been extensively reviewed elsewhere [16, 42, 43]. For pyrexia treatment, choices between methods have not yet been determined on the basis of robust evidence but rather according to clinical criteria (listed in Table 2).

**Table 2:** Proposed criteria for choosing between pharmacological and non-pharmacological antipyretic methods

Antipyretic agents	Physical cooling
• Non sedated patients • Concomitant need for analgesia	• Hypothalamic dysfunction • Need for rapid induction • Need for strict temperature control • Patients with hemodynamic instability • Failure of antipyretic agents

#### Pharmacological methods

##### Paracetamol

Paracetamol is the most commonly administered antipyretic in clinical practice [44]. Compared with placebo or no treatment, the difference in body temperature usually reaches statistical significance, although this is modest with uncertain clinical significance. In patients with brain injury, a standard dose (3 g/day) of paracetamol is often reported as ineffective [16]. This justified increasing the dose to 6 g/day, i.e., above the recommended maximal daily dose of 4 g. This higher dose was shown to reduce body temperature by 0.3 °C within 4 h compared with placebo [45]. In the “PAIS” trial, 6 g/day paracetamol administered by the enteral route in patients with stroke resulted in a mean body temperature significantly lower than with placebo [35]. This difference was limited to 0.26 °C (95 % CI 0.18–0.31) at 24 h. Of note, this study did not find any improved outcome with paracetamol. Recently, a pilot study in TBI failed to show a significant reduction in core body temperature despite the use of 6 g/day intravenous paracetamol [46]. The combination of 1 g paracetamol and 800 mg ibuprofen was tested for its ability to control fever in 79 neurological ICU patients [47]. Temperature lowering was enhanced by the combined treatment compared with patients who received paracetamol alone.

In the “HEAT” trial performed in sepsis, the efficacy of 4 g/day intravenous paracetamol was disappointing compared with placebo [29]. Whilst statistically significant within the first three days of treatment, the maximum difference between mean daily temperatures was recorded on day 1, with a between group difference of 0.48 °C (95 % CI −0.59 to −0.36), only. This modest difference may be related to the lack of paracetamol’s efficacy or the rapid spontaneous normalisation of temperature in the placebo group. The negative result of this study could be explained by insufficient difference in temperatures. In addition to its antipyretic properties, paracetamol is an antioxidant. In a placebo-controlled phase II trial including 40 patients with severe sepsis, a reduction in oxidative stress related to cell-free haemoglobin was found with paracetamol [48]. All these recent trials show that paracetamol is well tolerated when patients with liver dysfunction are excluded. The safety of paracetamol remains to be evaluated in patients at higher risk of ischemic liver failure and with hypotension.

##### Non-steroidal anti-inflammatory agents (NSAIDs)

NSAIDs are regularly used in the ICU despite the lack of adequate safety evaluation. NSAIDs have a well known side effect profile including hypotension, impaired hepatic and renal function, sodium and water retention, gastrointestinal bleeding and platelet dysfunction. In an attempt to avoid some of these effects, low dose continuous infusion of diclofenac has been proposed. In a small RCT, a low dose infusion was sufficient to control fever in patients with brain injury with fewer episodes of pyrexia compared with the standard bolus dosing group [49]. In a RCT including 79 neurological ICU patients, a similar temperature profile was found after a single dose of ibuprofen compared with paracetamol [47]. In sepsis, NSAIDs have been tested for their ability to modulate the inflammatory response [15, 50]. Although fever was not an inclusion criterion, an antipyretic effect was observed compared with placebo. In 40 patients treated with loraxicam, the maximum between-group difference in temperature was ≈0.6 °C after 24 h of treatment [50]. In the landmark study on ibuprofen, a NSAID allowed a more rapid decrease in temperature with a maximal between-group difference of ≈0.9 °C [15]. Similar outcomes and adverse effects were observed with NSAIDs and placebo. Nevertheless, NSAID use should be discouraged in sepsis until further safety evaluations have been performed. NSAIDs are clearly a risk for worsening the evolution of severe infections [51, 52].

#### Non-pharmacological methods

Various surface and endovascular automatic cooling devices allowing tight temperature control are now available [42]. When used with the aim of normothermia induction and maintenance, the main advantage of automatic devices is the avoidance of hypothermia. Automatic devices are more expensive but reduce the nursing workload.

##### Surface cooling devices

Three main types of surface cooling devices are available: air-circulating blankets, water circulating blankets and hydrogel-coated water-circulating pads [42]. There is no evidence to support the use of fans for temperature control. Fans are usually considered to help with patient comfort but they can induce shivering [42].

In febrile ICU patients, air-circulating blankets seem less effective for the induction of normothermia compared with the other surface cooling devices [53]. For the maintenance of normothermia, all surface cooling devices were equivalent [53]. Opposite results showing better control using air-circulating blankets were found in two smaller studies [1, 54]. In a RCT including 53 neurological ICU patients, water-circulating pads showed a significantly more rapid induction of normothermia with better control compared with conventional water-cooling blankets [55]. Shivering occurred more frequently with pads (39 versus 8 %). The tolerance of all surface cooling devices appears to be acceptable with very few skin injury complications reported.

##### Endovascular cooling devices

Several intravenous heat exchange catheter devices are available for temperature management [42]. Endovascular cooling was initially evaluated for therapeutic hypothermia. Some controlled studies are now available in patients with acute brain injury managed with controlled normothermia. The obvious disadvantage is their associated risks, which are likely similar to those associated with invasive central vascular access.

In 296 neurological ICU patients randomized to receive fever treatment either by heat exchange catheter or by paracetamol plus cooling blanket, the burden of fever was significantly reduced with the use of endovascular cooling with no more adverse events [56]. The occurrence of shivering was rare (3.7 %) but of note all patients were ventilated and sedated. A RCT including 102 patients with cerebrovascular disease also demonstrated a significant reduction in fever burden with endovascular cooling compared with a NSAID plus water-circulating blanket [21]. The overall incidence of infection was significantly higher with endovascular cooling compared with an antipyretic and surface cooling. Whether this was related to the invasive device or, finally, to better control of pyrexia with decreased host defences needs to be studied further.

Renal replacement therapies are not typically indicated for temperature control but, in patients requiring renal support, they contribute to heat loss and participate in pyrexia control. Negative heat balance may improve hemodynamic tolerance through better vascular tone [57]. Renal replacement therapies may represent a confounding factor in comparative trials on temperature control.

##### Thermal tolerance of cooling

Any decrease in core and/or peripheral temperature will result in vasoconstriction followed by shivering. In normal and febrile conditions, shivering commences at a body core temperature of ≈1.5 °C under the hypothalamic set point [58]. Skin temperature accounts for around 20 % of thermoregulation and cold stress can promote shivering while the core temperature remains constant [59]. Some studies report less shivering with endovascular cooling but the results are inconsistent [42].

Cooling patients with an elevated temperature set point will promote the shivering reflex to produce heat and counter core temperature lowering. Shivering not only impedes thermal control but its metabolic cost is substantial [60, 61]. Cooling awake septic patients increases VO_2_ by up to 60 % [61]. Shivering also promotes the cardiovascular and respiratory stress response and increases cerebral metabolic stress. Avoidance of shivering is, therefore, a crucial component of the cooling procedure. The administration of an antipyretic agent to reduce the temperature set point before commencement of cooling is a common practice but appears to be ineffective [60, 61].

Pharmacological and non-pharmacological management of shivering has been proposed [16, 43]. Given the indication for cooling, many of these disease processes occur in patients who are already receiving some form of sedation. Slight anaesthesia decreases the shivering threshold and represents the most efficient way to prevent it and achieve the goal of VO_2_ and cardiovascular stress reduction [13, 14, 27]. In awake patients, the benefit of pyrexia treatment using cooling should be clearly evaluated against the risk of metabolic and cerebral stress induced by shivering, especially given that shivering can occur without any clinical manifestation and may only be detected by VO_2_ monitoring [60].

#### Pharmacological versus non-pharmacological methods

A meta-analysis of 11 trials considered pharmacological versus non-pharmacological antipyretic treatments with outcome measures being targeted temperature and haemodynamic effects [62]. It found that intravascular as opposed to surface cooling had better target temperature results, although there was a non-significant trend towards higher mortality. Only three small studies consisted of a head-to-head comparison of pharmacologic and non-pharmacologic methods, for which the analysis was inconclusive [62].

In sepsis, the three largest RCTs compared ibuprofen [15], paracetamol [29] and surface cooling [27] against placebo or no treatment. The maximal between-group differences in temperatures reported were 0.6 °C on day 1, 0.9 °C at 10 h and 1.6 °C at 12 h, respectively. Although inconclusive, these data may suggest that controlling fever by surface cooling is more efficient than by antipyretic agents.

## Conclusions

There is now awareness that a balance is required between the severe metabolic stress induced by pyrexia and its possible contribution to host defences. On what side the balance is can strongly vary between patient groups. The precise, safe and efficient control of temperature is now well within our ability, although analysis of the literature does not provide recommendations for preferred methods of treatment in clinical practice. Several studies have found certain techniques have some superiority over others but none have demonstrated a beneficial clinical impact of a more rapid induction or a better control of normothermia on patient outcome. Further studies are needed to determine which patients would benefit the most from control of pyrexia and by which means this should be implemented.

## Abbreviations

CI: Confidence interval
ICU: Intensive care unit
OHCA: Out-of-hospital cardiac arrest
NSAID: Non-steroidal anti-inflammatory drug
RCT: Randomised controlled trial
TBI: traumatic brain injury
TTM: Targeted temperature management
VCO_2_: Rate of elimination of carbon dioxide
VO_2_: Rate of oxygen consumption
